# P-2219. Demonstrating a Novel Immune Cell Isolation Strategy for Scalable Single-Cell RNA Sequencing Studies in Patients with Sepsis

**DOI:** 10.1093/ofid/ofae631.2373

**Published:** 2025-01-29

**Authors:** Alyssa DuBois, Pierre Ankomah, Michael R Filbin, Roby P Bhattacharyya

**Affiliations:** Broad Institute, Boston, Massachusetts; Massachusetts General Hospital, Boston, Massachusetts; Massachusetts General Hospital, Boston, Massachusetts; Massachusetts General Hospital, Boston, Massachusetts

## Abstract

**Background:**

Though immune dysregulation often plays a key role in disease, characterizing circulating immune cells in critical illnesses is challenging. Studies using single-cell RNA sequencing (scRNA-seq) of peripheral blood mononuclear cells (PBMCs) reveals the type and function of patients' immune cells (cell substates) that could advance diagnostics and therapeutics, particularly in diseases defined by immune response heterogeneity such as sepsis. However, PBMC isolation from blood is required for scRNA-seq, and the gold-standard method (density gradient separation, or ‘Ficoll’) requires two hours of onsite processing, precluding large-scale sample collection at most clinical sites. Simplifying sample collection could enable participation of more health care centers, as well as the over-enrollment of patients for later retrospective adjudication, better reflecting the true patient heterogeneity in diseases.

**Figure 1.**
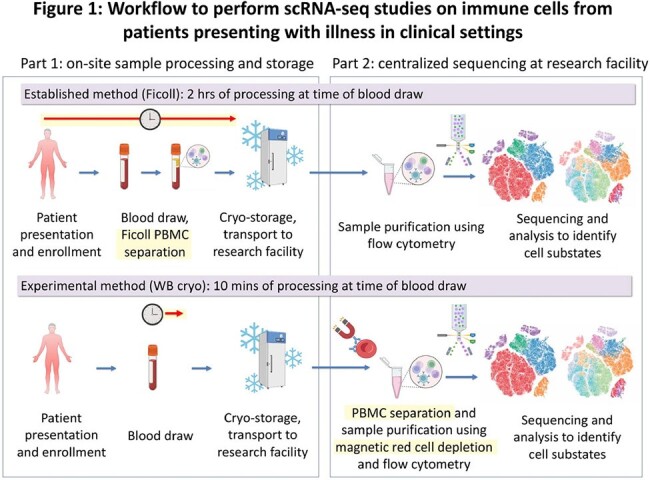
Workflow depicting the process from patient presentation in a health care center to sequencing results. The WB cryo method expedites sample collection in clinical settings by eliminating the need for Ficoll PBMC separation, a time-intensive process requiring laboratory equipment and trained operators. Differences in methods are highlighted in yellow.

**Methods:**

We developed an alternative PBMC isolation method from cryopreserved whole blood (WB-cryo) using magnetic- and fluorescence- activated cell sorting (Fig. 1). We isolated PBMCs using the WB-cryo and Ficoll methods from patients with sepsis (n = 5) who sought evaluation at an Emergency Department and one healthy control. We performed scRNA-seq to identify and compare cell substate proportions and marker genes for key substates.

**Figure 2.**
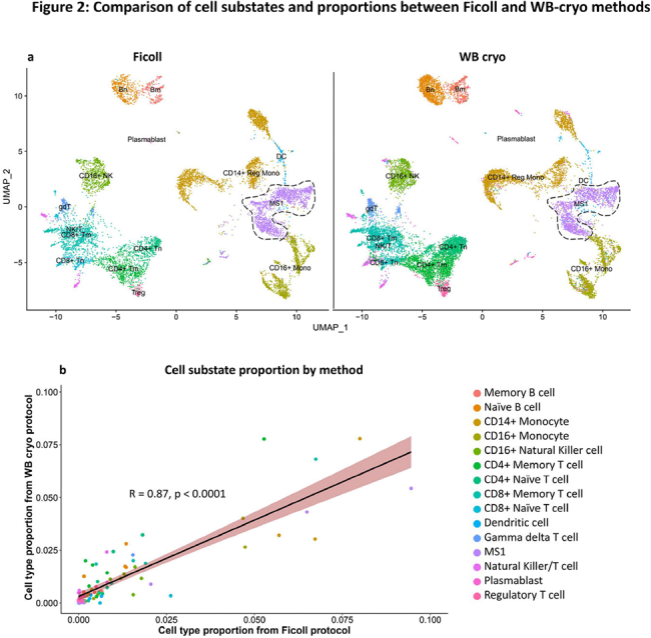
Results of scRNA-seq on PBMCs isolated using Ficoll and WB cryo methods (n = 5 patients with sepsis, 1 healthy control). a) Uniform Manifold Approximation and Projection (UMAP) of separately clustered and annotated scRNA-seq data from each method; Bm denotes memory B cells, Bn: naive B cells, NK: natural killer cells, Mono: monocytes, MS1: monocyte substate 1, DC: dendritic cells, Tn: naive T cells, Tm: memory T cells, gdT: gamma delta T cells, Treg: regulatory T cells. b) correlation plot of resulting cell substate proportions (R = 0.87, p < 0.0001).

**Results:**

Analysis included 9,996 cells isolated by Ficoll and 15,482 cells from WB-cryo. Sequencing quality was comparable between methods, as were cell substate fractions (Pearson correlation 0.87, p < 0.0001) (Fig. 2), and marker genes for a key monocyte substate enriched in patients with sepsis (19 of top 20 marker genes shared). WB-cryo reduced onsite sample processing time from 2 hours to < 10 minutes.

**Conclusion:**

PBMCs isolated by WB-cryo retained similar transcriptional states when compared to PBMCs isolated using Ficoll, with > 10-fold less onsite hands-on time. With further validation, WB-cryo may expand scRNA-seq study enrollment in sepsis and other diseases. Ongoing work in a larger cohort (n = 23 patients with sepsis) processed across two clinical sites is underway to validate our isolation method as a way to extend the utility of scRNA-seq in multisite studies of complex and heterogeneous conditions like sepsis.

**Disclosures:**

Michael R. Filbin, MD, Day Zero Diagnostics: Grant/Research Support|Quidel: Grant/Research Support

